# Effect of calcium carbonate nanoparticles, silver nanoparticles and advanced platelet-rich fibrin for enhancing bone healing in a rabbit model

**DOI:** 10.1038/s41598-023-42292-x

**Published:** 2023-09-14

**Authors:** Mohamed Abd-Elkawi, Ahmed Sharshar, Tarek Misk, Islam Elgohary, Shaaban Gadallah

**Affiliations:** 1 Department of Surgery, Radiology and Anesthesiology, Faculty of Veterinary Medicine, New Valley University, Alkharga, New Valley 2715 Egypt; 2 Department of Surgery, Radiology and Anesthesiology, Faculty of Veterinary Medicine, University of Sadat City, Sadat City, Egypt; 3 Department of Pathology, Agricultural Research Center (ARC), Animal Health Research Institute (AHRI), Eldokki, Giza, Egypt

**Keywords:** Biotechnology, Cell biology, Medical research, Materials science, Nanoscience and technology

## Abstract

This study aimed to evaluate the efficacy of calcium carbonate nanoparticles (CCNPs) to induce new bone formation in a critical size segmental bone defect in rabbit’s radius when used alone, combined with silver nanoparticles (AgNPs) as a paste, or as a composite containing CCNPs, AgNPs, and advanced platelet-rich fibrin (A-PRF). Thirty-six adult apparently healthy male New Zealand White rabbits aging from 5 to 6 months and weighting 3.5 ± 0.5 kg were used. The animals were divided into four groups; control group, CCNPs group, CCNPs/AgNPs paste group, and CCNPs/AgNPs/A-PRF composite group. The animals were investigated at 4, 8, and 12 weeks post-implantation in which the healing was evaluated using computed tomographic (CT) and histopathological evaluation. The results revealed that CCNPs/AgNPs paste and CCNPs/AgNPs/A-PRF composite has a superior effect regarding the amount and the quality of the newly formed bone compared to the control and the CCNPs alone. In conclusion, addition of AgNPs and/or A-PRF to CCNPs has reduced its resorption rate and improved its osteogenic and osteoinductive properties.

## Introduction

Bone is biochemically and structurally complex. It has a unique capacity to regenerate and remodel itself without scar tissue formation under normal circumstances^1^. Bone healing is often impaired in many clinical situations especially when large bone defects are created as in cases of severe trauma and after tumor resection. In such cases a more sophisticated regeneration strategy is required to promote bone healing, and therefore improve its mechanical function^2^.

Over the past years, various synthetic and biologically derived bone graft materials have been evaluated for their ability to restore bone continuity. Each of them has its own advantages and disadvantages^3,4^. In recent years nanotechnology and tissue-engineering have occupied an important position among these materials^5^.

Nanomaterials are manufactured with a grain size less than 100 nm which behave very differently to their conventional counterparts with similar anatomical structure^6^. Nano ceramics is one of nanomaterials that has been used heavily in bone healing research recently, it mimics the hierarchical and nanoscale features of bones and it has been emerging as a new viable class of materials for bone fracture repair. It has shown improved bone cell functions compared to their conventional counterparts^6,7^.

Comparing to other nanoparticles, Calcium carbonate nanoparticles (CCNPs) and silver nanoparticles (AgNPs) have received a great attention as a bone substitute^8^. CCNPs showed high flexibility in preparation, tailoring, biodegradation, and osteoconductivity. It has also a higher degradation rate compared with other synthetic bone substitutive ceramics, including nano-β-tricalcium phosphate and nanohydroxyapatite. Along with it can enhance gene expression in specific osteogenic markers and induce osteoblast differentiation and proliferation^9–11^.

AgNPs are characterized by exclusive biological, chemical, and physical properties in comparison to their large-size equivalents. It possesses a respectable antibacterial property against different microorganisms. It encourages the formation of fibrous joint and the subsequent end joining of the fractured ends^12–14^. AgNPs also promote osteogenesis by upregulation of different bone morphogenic proteins (BMPs)^15^.

Platelet-rich fibrin (PRF) is a second-generation platelet concentrate consisting of fibrin clot rich in platelets, cytokines, leukocytes, and circulating stem cells which are effective in bone regeneration^16^. PRF membrane has a very significant slow and continuous secretion of critical growth factors up to 28 days which offers a great potential for wound healing^17^. Ghanaati^18^ and his co-workers performed a new protocol for obtaining a new product of PRF called A-PRF by centrifugation of the whole blood at slower speeds for a longer period. It supports significantly higher growth factor release over a longer period compared to standard PRF and proves clinically beneficial regenerative procedures^19,20^. As the best as we know, there is no study has discussed the use of CCNPs as a filling biomaterial either alone or combined with AgNPs and A-PRF for long bone critical segmental bone defect. Therefore, this study aimed to evaluate the efficacy of CCNPs to induce new bone formation in a critical size segmental bone defect in rabbit’s radius when used alone or combined with AgNPs as a paste, or as a composite containing CCNPs, AgNPs, and A-PRF.

## Materials and methods

### Animal model and housing

Animals were managed according to the guide for care and use of laboratory animals approved by the Animal Care and Use Committee, University of Sadat City–faculty of Veterinary Medicine, Sadat City, Egypt (registration number: VUSC-031-1-21) and was carried out under relevant guidelines and regulations. This work was done in compliance with the ARRIVE guidelines and regulations (https://arriveguidelines.org).

All animals have been accommodated and cared for according to the Egyptian animal welfare law (No. 53, 1966).

Thirty-six apparently healthy adult male New Zealand White rabbits were used in this study. the mean age of the animals was 6 ± 1 months and its weights ranging from 3 to 4 kg. The animals were housed singly in stainless steel cages and were kept in humidity 60% with 12 h light/dark cycle and a temperature of 23 (± 2)°C. They had free access to standard diet and water during the whole period of the experiment. All rabbits were vaccinated against Rabbit Hemorrhagic Disease virus (RHDV) using (0.5 ml s/c, bivalent RHDV gel vaccine®, SERVAC Co.; A.R.E) and protected against external and internal parasites using (Dectomax®, Zeotis Co.; A.R.E). The experimented rabbits were randomly and equally divided into four groups (n = 9), each group equally divided into three subgroup according to the observation period 4th, 8th and 12th week (n = 3).

### Bone substitutes preparation

CCNPs and AgNPs were synthesized in the Nanomaterials Synthesis and Research unit, Animal Health Research Institute (AHRI), Agriculture Research Centre (ARC), Egypt.

Following the instructions of *Hussein et al.*^21^ in preparing CCNPs and *Amer et al.*^22^ for preparing AgNPs.

### Characterization of the prepared nanoparticles

1. Dynamic Light Scattering (DLS)

DLS showed that the average particle size of CCNPs was 88 nm while the average particle size of AgNPs was 10.4 nm.

2. Transmission Electron Microscope (TEM)

CCNPS appeared as monodisperse, un agglomerated nanospheres while AgNPs appeared as ultrafine uniform, well dispersed spherical nanoparticles.

3. X-ray diffraction (XRD)

The phase identification and structure of the CCNPs in which the presence of CCNPs can be observed at 20 of 23.0, 29.5, 36.0°.

A-PRF membrane was prepared just before the implantation according to *Ghanaati et al.*^18^. It has been prepared by obtaining 5 ml of whole blood from the central ear vein. The blood was centrifuged at 1500 rpm for 14 min. The formed PRF clot was dissected from RBCs and was compressed between two sterilized gauzes. CCNPs/AgNPs paste CCNPs/AgNPs/A-PRF composite were prepared on the same basis as described by *Abd Elkawi et al.*^23^.

### Surgical procedure

The mid-shaft of the right radius was exposed via 5 cm cranial skin incision at the fore arm. After the muscles were dissected, a 14 mm segmental defect was made using an electric saw. The defect was left empty in the control group (group A), filled with CCNPs paste in group (B), in group (C), the defect was reconstructed with CCNPs/AgNPs paste, while in in group (D), it was reconstructed with CCNPs/AgNPs/A-PRF composite.

### Radiographic evaluation

The operated radius was scanned using a spiral-computed tomography (CT) multidetector at the 4th, 8th, and 12th week post-operatively using a middle-frequency kernel, 0.9- mm-thick axial images. The obtained images were undergone three-dimensional (3D) multiplanar reconstruction using the RadiAnt DICOM image processing software (Medixant Co, Poznan, Poland). The quantitative bone densities at the operation site (the area of interest, AOI) and the adjacent bone (BAD) were also measured and correlated.

#### Histo-pathological evaluation

At each time point 4, 8, and 12 weeks, thirty-six rabbit were euthanized following the guideline of the American Veterinary Medical Association (AVMA), Euthanasia was done by injecting the rabbit with xylazine Hcl at dose 5 mg/Kg for sedation followed by thiopental sodium injection at dose 100 mg/Kg intravenously. Two cm bone specimens were harvested and fixed immediately in 10% buffered formalin for 72 h. The samples were decalcified using 10% EDTA di sodium for one month. Sections of 5 μm thickness were obtained, stained with Hematoxylin & Eosin stain, and covered with coverslips. The defects were evaluated by two pathologists using the Emery scoring system^24^ (Table 1).

**Table 1:** Histological scoring standard by *Emery* et al.^24^.

Item	Score
Empty gap	0
Filled with fibrous connective tissue only	1
More fibrous tissue than fibrocartilage	2
More fibrocartilage than fibrous tissue	3
Fibrocartilage only	4
More fibrocartilage than bone	5
More bone than fibrocartilage	6
Filled only with bone	7

#### Statistical analysis

The obtained values were reported as median, and standard deviations. A one-way ANOVA was used to carry out statistical analysis *p* ≤ 0.05 was considered statistically significant. All obtained values were analyzed using the SPSS software (version 20.0; IBM, America).

## Results

**Figure 1:**
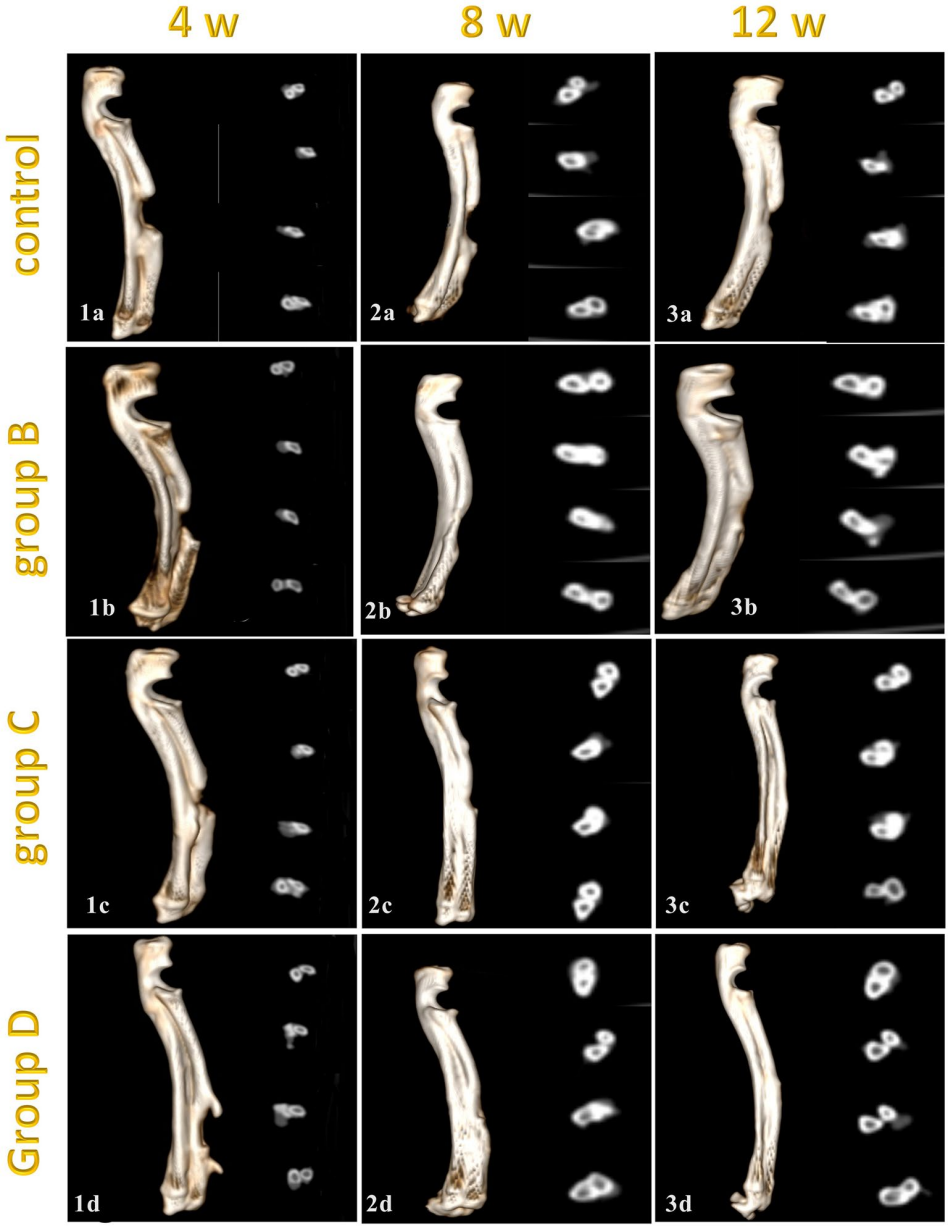
Computed tomography 3D images (Right) and cross sections images (left) in control (**a**), CCNPs (**b**), CCNPs/AgNPs (**c**) and CCNPs/AgNPs/A-PRF (**d**) at 4 weeks (1), 8 weeks (2) and 12 weeks (3) post implantation.

Concerning the 3D images and the axial plane cross sections, the control group showed an empty defect at 4- and 8-weeks post-implantation with a few amounts of new bone in-growth without the appearance of any signs of remodeling (canalization) at the end of the study. On the other hand, the other groups showed new bone in-growth at 4- and 8-weeks post-implantation with various degrees of defect filling and bone remodeling. At the end of the study group C and group D showed complete defect filling with new bone formation but only group D showed complete canalization (Fig. 1).

**Figure 2:**
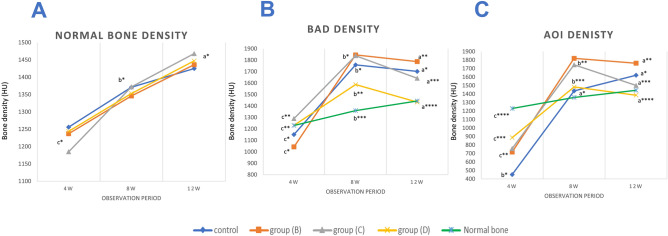
Showing (**A**) the normal density of the radius of the four experimental groups. It showed a marked increase with animal age with no significant difference among the experimental groups at each evaluation time-point (*P* = 0.766–0.937). (**B**) the AOI density of all examined groups at different time points (4,8, and 12 weeks). There is a significant difference between all groups (*P* = 0.000) throughout all time points (*P* = 0.000). At the end of the observation period the AOI density of group (D) (1386) was very close to the normal bone density (1444.3) followed by group (C) (1501). (**C**) Showing the BAD density of all examined groups at different time points (4,8, and 12 weeks). There is a significant difference between all groups (*P* = 0.001–0.004) throughout all time points (*P* = 0.000). As shown in the results group (D) had relatively least bone density and at the end of the observation period the BAD density (1434) was relatively the same as the normal bone density (1444.3).

**Figure 3:**
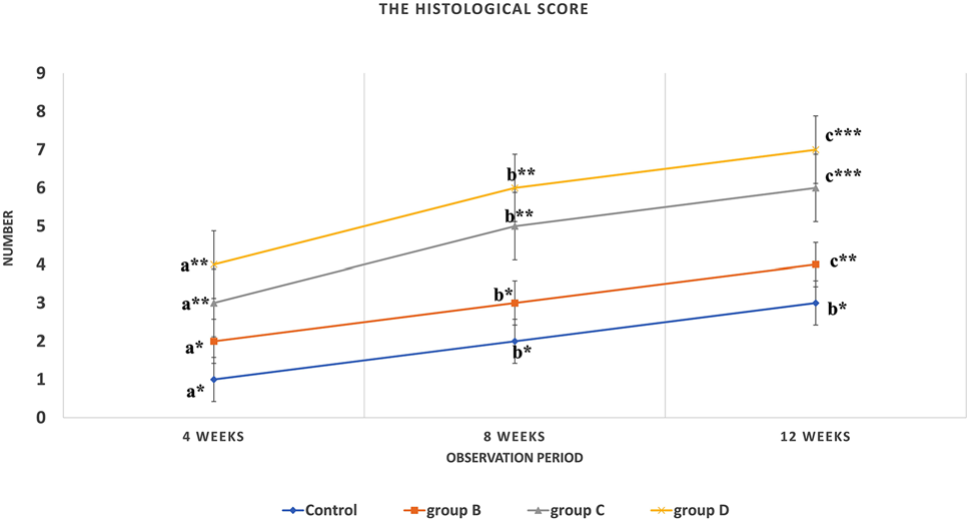
The histopathological score showed great significant differences in the histopathological score at 4,8 and 12 weeks of the observation period among the four experimental groups (*P* = 0.000). At the end of the observation period (12 weeks), group B couldn't make complete healing although the presence of some newly formed bone, while group C&D showed complete healing with score = 6–7 respectively at the end of the observation period with a statistical difference (*) between them versus the two other groups (group B and control).

The results of the quantitative measurements of bone densities showed significant deference in normal radial densities in relation to different time points throughout the study (*P* = 0.000–0.031). While no significant difference was reported among the four experimental groups at the same observation period (*P* = 0.766–0.937). There was a significant difference in AOI density between all groups (*P* = 0.000) at all time points (*P* = 0.000). The AOI, group D showed relatively low density compared to group B and C. At the end of the observation period, the AOI density of group D was (1386 HU) which is very close to the normal bone density (1444.3 HU) followed by group C (1501 HU). The control group showed the least AOI density (452.5 HU) among all tested groups and was found in the 4th post-operative week. Concerning BAD density, there was a significant difference in BAD density between all groups (*P* = 0.001–0.004) throughout all time points (*P* = 0.000) (Fig. 3). Group D had the least bone density between all examination groups. Which become (1434 HU) close the normal bone density by the end of the observation period (1444.3 HU) followed by group C (Table 2) (Fig. 2).

**Table 2:** Results of quantitative bone densities of normal bone, new bone in growth (AOI density) and density of bone adjacent to the defect area (BAD density) by CT and expressed as mean ± SD.

Groups	4w	8w	12w	*P*-value
Normal bone density				
Control	(1256.6 ± 39.7)^c^*	(1371 ± 26.8)^b^*	(1425.33 ± 56.2)^a^*	0.000
CCNPs	(1237.3 ± 122.2)^c^*	(1346 ± 41.5)^b^*	(1436.3 ± 83.6)^a^*	0.01
CCNPs/AgNPs	(1185.6 ± 112.1)^c^*	(1372.3 ± 47.4)^b^*	(1468 ± 55.02)^a^*	0.031
CCNPs/AgNPs/APRF	(1244 ± 241.79)^c^*	(1353 ± 29.8)^b^*	(1447.6 ± 65.1)^a^*	0.000
Total mean	1230.91 ± 129.2	1360.5 ± 33.3	1444.3 ± 58.67	0.000
*P*-value	0.937	0.766	0.874	
BAD density (bone adjacent to defect)				
Control	(1151 ± 117)^c^*	(1759 ± 57.98)^b^*	(1702 ± 78.49)^a^*	0.000
CCNPs	(1043 ± 50)^c^*	(1846 ± 75.66)^b^*	(1788 ± 16.97)^a^**	0.000
CCNPs/AgNPs	(1290 ± 10)^c^**	(1839 ± 55.14)^b^*	(1642 ± 18.67)^a^***	0.000
CCNPs/AgNPs/APRF	(1231 ± 54)^c^**	(1587 ± 64.38)^b^**	(1434 ± 21.60)^a^****	0.000
*p*-value	0.001	0.004	0.001	
AOI density (area of interest)				
Control	(452.5 ± 12.83)^b^*	(1366 ± 42.62)^a^*	(1306 ± 22.94)^a^*	0.000
CCNPs	(717.5 ± 19.77)^c^**	(1820 ± 60.30)^b^**	(1663 ± 14.65)^a^**	0.000
CCNPs/AgNPs	(758 ± 5.66)^c^**	(1744 ± 81.38)^b^**	(1501 ± 34.18)^a^***	0.000
CCNPs/AgNPs/APRF	(887 ± 4.37)^c^***	(1483 ± 29.08)^b^***	(1386 ± 11.73)^a^****	0.000
*P*-value	0.000	0.000	0.000	

The data showed a significant difference in AOI and BAD densities between all groups at all time points (*P* = 0.000–0.004).

*,**,***Medians and ranges with different asterisks superscripts in the same column are significantly different at *P* < 0.05.

^a,b,c^Medians and ranges with different small superscripts letters in the same row are significantly different at *P* < 0.05.

Histological evaluation of the bone defect showed the superiority of group (D) over other groups. In group (D), the maturity of the newly formed bone started as early as by the end of the 4th week and become completely mature by the end of the 8th week post-implantation. On the other hand, bone maturation retarded till the end of the 8th, and 12th week post-implantation in group (C) and group (B) respectively. The control group failed to show ossification and rather formed fibrocartilage after 12 weeks (Table 3) (Fig. 3).

**Table 3:** Showing the histological score evaluation of the four experimental groups at 4, 8, 12 weeks post operation.

Time (weeks)				
Groups	4w	8w	12w	*P*-value
Control	1(1–1)^a^*	2(2–3)^b^*	3(2–3)^b^*	0.001
CCNPs	2(1–2)^a^*	3(3–4)^b^*	4(4–5)^c^**	0.000
CCNPs/AgNPs	3(3–4)^a^**	5(5–6)^b^**	6(6–7)^c^***	0.000
CCNPs/AgNPs/APRF	4(3–4)^a^**	6(5–6)^b^**	7(7–7)^c^***	0.000
*P*-value	0.000	0.000	0.000	

The data showed a significant difference between all experimented groups at all time points (*P* = 0.000–0.001).

*,**,***Medians and ranges with different asterisks superscripts in the same column are significantly different at *P* < 0.05.

^a,b,c,d^Medians and ranges with different small superscripts letters in the same row are significantly different at *P* < 0.05.

**Figure 4:**
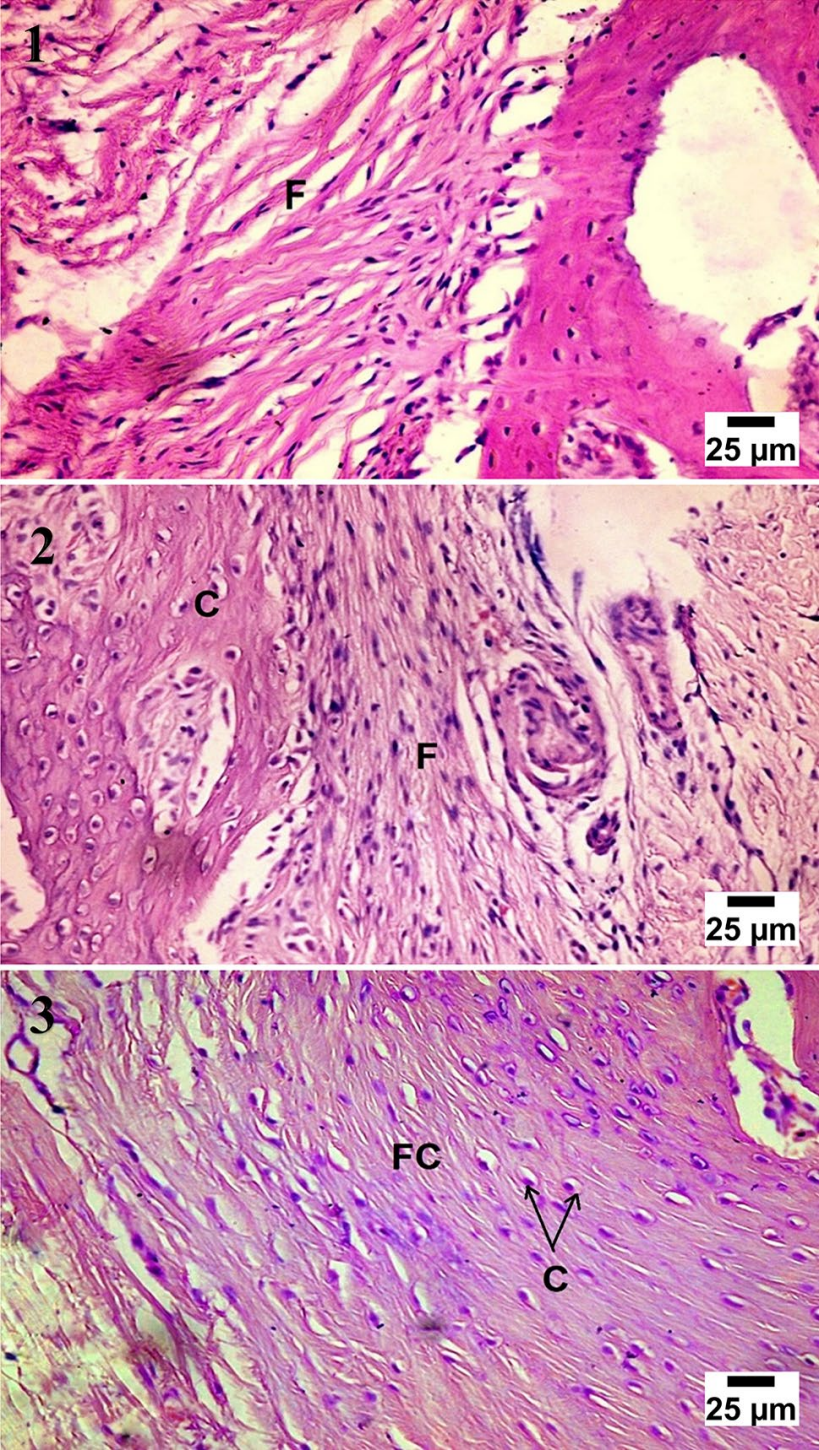
Microscopic appearance of the control group showed at 4 weeks post-operative (PO) (1) the defect filled with unorganized fibrous tissue (F) with a large number of chondroblasts and osteoblasts (black arrows) at the bony edges. At 8 weeks PO (2), the defect was filled with cartilaginous tissue formation (C) with mature chondrocytes within organized fibrous tissue (F) while at 12 weeks PO (3), the defect was filled with fibrocartilage (FC) characterized by mature chondrocytes (C) in separate lacunae within fibrous tissue bundles.

**Figure 5:**
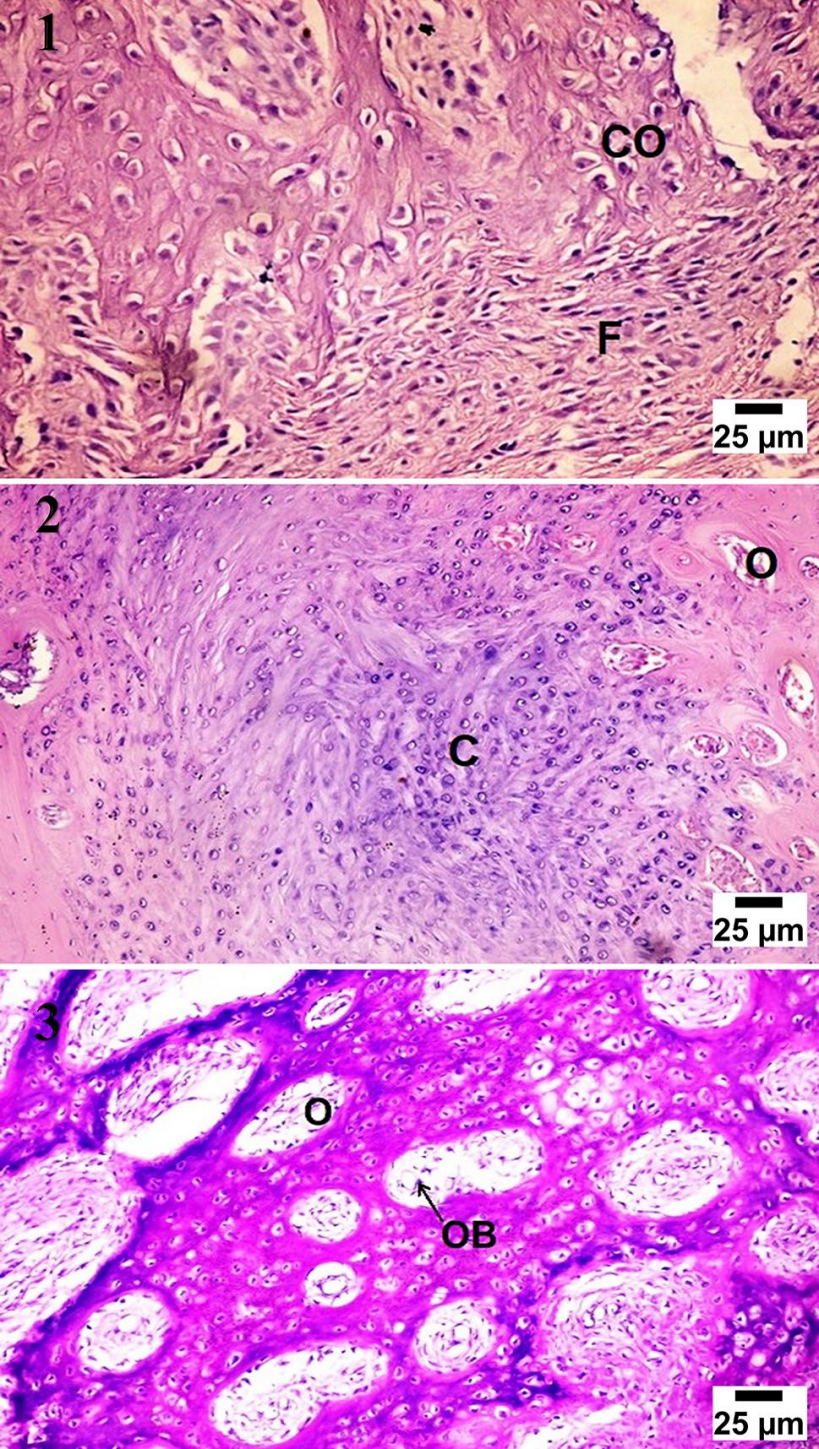
Microscopic appearance of CCNPs group (group B) showed at 4 weeks PO (1), the defect filled with the cartilaginous transition from fibrocartilage characterized by migration of a large number of chondrocytes (Co) in separate lacunae within the fibrous tissue bundles (F). At 8 weeks PO (2), the defect was filled with large hyaline cartilage (C) with few osteonal canals on the peripheries of the defect (O), while at 12 weeks PO (3), the defect showed an increase in osteonal canal (O) formation within the calcified cartilage with a high number of osteoblasts (OB) within the osteonal canals.

**Figure 6:**
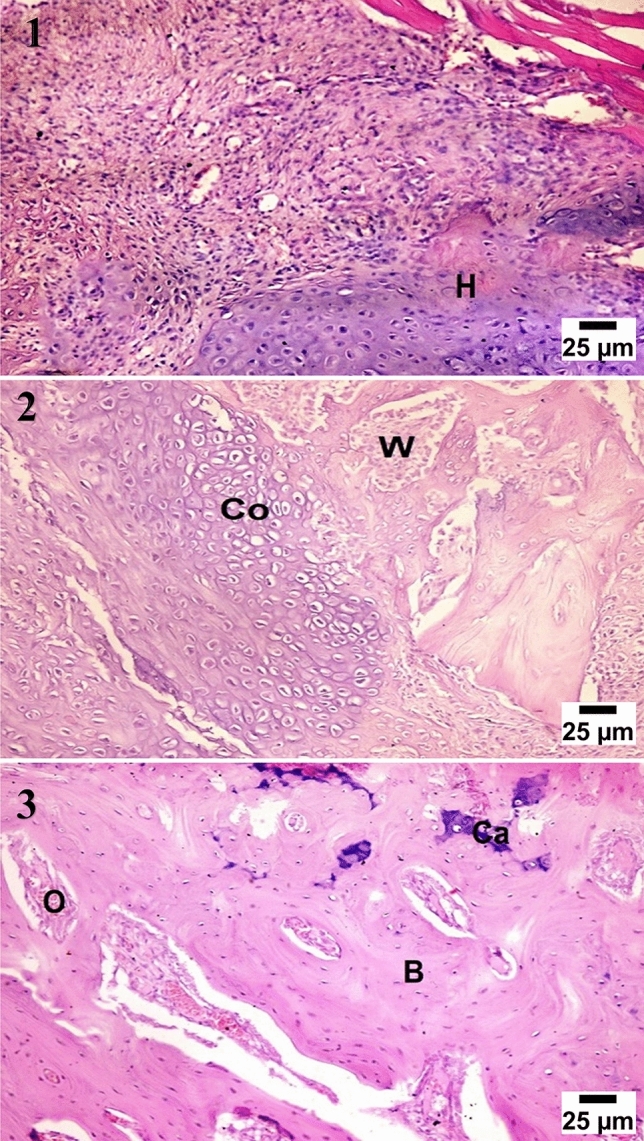
Microscopic appearance of CCNPs/AgNPs group (group C) showed at 4 weeks PO (1), the defect filled with cartilaginous transition phase with formation of hyaline cartilage (H) and multiple mature chondrocytes in lacunae. At 8 weeks PO (2), the defect showed formation of fibrous connective tissue and woven bone (W) migrating to replace hypertrophic chondrocytes (Co) at the cartilage bone junction, while at 12 weeks PO (3), the defect showed the formation of the haversian system from osteonal canals (O) and trabecular bone condensation into compact bone (B) with calcium deposition (Ca).

**Figure 7:**
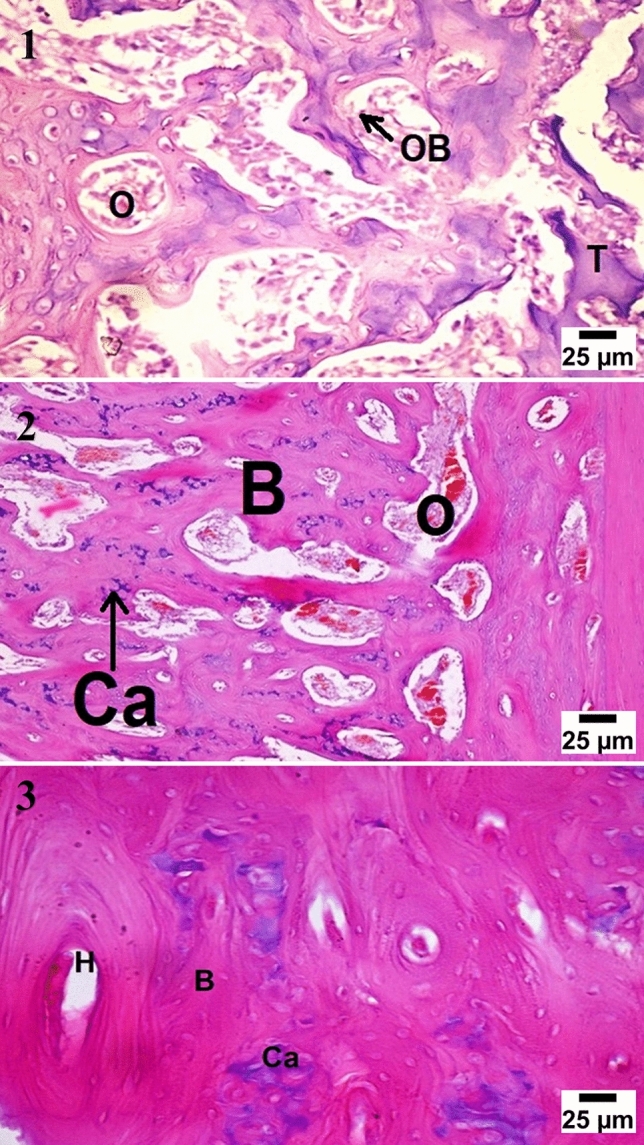
Microscopic appearance of CCNPs/AgNPs/A-PRF group (group D) showed at 4 weeks PO (1), the defect filled with multiple osteonal canals (O) formation with a large number of osteoblasts (OB) forming trabecular bone (T) with active calcium deposition. At 8 weeks PO (2), the defect showed multiple osteonal canals (O) with compact bone formation (B) and calcium precipitation (Ca), while at 12 weeks PO (3), the defect showed the formation of the haversian system (H) with ossification and dense compact bone formation (B) with calcium deposition (Ca).

At four weeks observation period, histological examination showed cartilaginous transformation and appearance of the mature chondrocytes in most of the treated groups, the control group showed unorganized fibrous tissue filling the center of the defects with a large number of chondroblasts and osteoblasts at the bony edges. At eight weeks observation period, significant bone growth was seen in group (D) forming more dense compact bone, and group (C) showed large ossification centers expressed in osteonal canals with hypercellularity with osteoblasts, the groups (B) showed very few osteonal canal formation at the periphery of the defect. At twelve weeks observation period, group (D) and group (C) showed more condensation of the bone structure and calcium deposition which was higher in group (D), the groups (B) showed an increase of osteonal canals within the cartilaginous tissue with a high number of osteoblasts (Figs. 4, 5, 6, 7).

## Discussion

Management of large bone defects; particularly that resulted from infection, severe trauma, resection of tumors or cysts, or that resulted from the failure of treatment such as delayed or non-union presents a great challenge for veterinary orthopedists^25^. Different techniques have been established for the reconstruction of bone loss and overcoming these problems^26^. Modern techniques are based mainly upon the improvement of bone regenerative capability which can be achieved by using cells capable of bone formation when delivered alone or with an osteoconductive scaffold to a skeletal defect^27^, or by using biologically active materials to overcome the inherent limitations associated with the use of autografts, allografts, and xenografts^28^.

A-PRF is widely used as a bone substitute. It’s known for its good bone-regenerating capacity^16^. As a fibrin biomaterial A-PRF contains the favorable components present in blood samples such as a large number of platelets, cytokines, and leukocytes^29^. There are many advantages of using PRF (the second generation of concentrated platelets) instead of PRP (the first generation) in bone healing. Preparation PRF does not need an anticoagulant (which interferes with the healing process), also it is prepared by one-step centrifugation at room temperature compared to PRP which needs two-steps centrifugation. Also, PRF gradually releases its growth factor content for a long period offering a continuous supply during the healing process, while PRP releases about 80% of its GFs content in the first 24 h^30^.

CCNPs is extensively used in drug delivery system. It is easily prepared, cost-effective, and had no toxic effect on animal models^31^. Although it has structure resembles the mineral structure of a normal bone, There is not enough data to discuss its role in accelerating bone healing^32^.

AgNPs are well known for its antibacterial, antifungal, antiviral, anti-inflammatory, and good osteogenic activities^33,34^. It is also, available, easily prepared, and cost effective^35^. Its use in the field of bone regeneration was limited in invitro application with calcium carbonate to reduce the prevalence of infection^36^. To our knowledge, this may be the first report which evaluated and described the in-vivo bone regenerating capacity of CCNPs when used alone and in combination with AgNPs and A-PRF to fill critical size bone defects in a rabbit model.

In our previous work, the prepared CCNPs and AgNPs biomaterials have been evaluated using DLS, TEM, and XRD examination. Our results revealed that CCNPs appeared as agglomerated spheres with 88.4 nm average particle size, with a characteristic peak positioned 20–29.5 at a wavelength of 1.54060. While AgNPs appeared as ultrafine uniform, well-dispersed spherical nanoparticles with 10.4 nm average particle size^23^. These results matched well with the results of Hill et al.^37^, which confirmed the nano-scale size of the used nanomaterials in this study.

The age of the used experimental animals, might be an important factor affecting the behavior of the examined biomaterials. In the present study, 6-month-old rabbits were included. Although long bones have reached about 95% of their adult length in rabbits by 16 weeks of age^33^, the skeletal growth of New Zealand White Rabbits complete between 19 and 32 weeks. In view of this, the use of rabbits older than 6 months should be recommended for bone regenerative studies^38^. The implantation time also plays a role as well. Some reports showed nonbridging of rabbit radial defects at 4–8 weeks of implantation^39^. In this study the observation period extended to 12 weeks. Our radiographic and histopathologic results showed that this period was sufficient for complete healing of bone defect. These result was found in accordance with^33,38^The obtained results of the quantitative analysis of the normal bone densities at different observation periods revealed no significant difference among the four experimental groups at each time point, However, it showed a significant difference at different time points. These data agreed with^40,41^, the authors stated that age was weakly but positively associated with cortical density at the tibial diaphysis among the healthy rabbits in which bone mineral density increases during skeletal maturation and continues four to eight weeks after the closure of the growth plates.

BAD and AOI densities values could be indicative of the resorption rate and pattern of the grafted material, the amount, and the quality of the newly formed bone. By tracking BAD and AOI densities curves throughout the study period, it has been noticed that BAD and AOI densities levels showed a marked reduction by the end of the 4th week as a result of active bone reaction at the defect periphery accompanied by resorption of the grafted materials. Group (D) showed the least value compared to other groups. This in turn supports the hypothesis that the addition of A-PRF to CCNPs/AgNPs paste greatly reduced its resorption rate. Throughout the rest of the observation period (8–12 weeks), both variables showed a gradual increase indicating active new bone formation to reach its maximum level by the end of the 8th week in all tested groups compared to the control. The same data was obtained by *Ou, et al*.^42^, the authors noticed that the density increased with healing duration in each group, which can be attributed to mineral deposition. After the end of the 8th week, both variables showed a gradual reduction in all groups compared to the control which indicates bone remodeling. At the end of the observation period, BAD and AOI densities were approximately equal to the normal bone density in group (C) and group (D) which indicate complete bone remodeling.

Our histological findings confirmed the former radiographic observation. It showed a marked increase of osteogenesis with new bone formation in groups (B), (C) and (D) compared to the control one which failed to show ossification and rather formed a fibrocartilage after 12 weeks. Osteogenesis and new bone formation in group (D) started by the end of the 4th week and became mature by the end of the 8th-week post-operation compared to other groups. This can be attributed to the improved bone cellular functions of the used nanomaterials compared to their micron-sized counterparts. As well as, the biological role of blood derivative (A-PRF) in the stimulation of neo-vascularization, and cellular migration. These data agreed with that reported by^37,43,44^ whom found that using autologous PRF in the induced gap has benefits for organizing the formative cell (especially osteoblast), formation of neovascularization and more rapid and faster apposition of the bony matrix with its mineralization process without significant inflammatory response, necrosis, or foreign body reactions demonstrating good osteoconductive and biocompatibility of enriched graft. This better healing potential noticed could be attributed to the osteoinductive effect of growth factors^45,46^.

In the same context, the addition of AgNPs to CCNPs showed improved osteogenesis of the implanted graft in which the defect was filled by new compacted bone with signs of remodeling and calcium deposition at the end of the observation period. These data are a confirmation of the study carried out by *Zhang et al*.^47^ and *Kumar et al*.^8^ in which the authors reported that AgNPs’ has proliferative and osteogenic differentiation induction effects on MSCs and promotion of fracture healing in the osteogenesis of mouse model. They stated that AgNPs could promote the formation of the fibrous joint and subsequently end joining of the fractured bone via multiple routes: chemo-attraction of MSCs and fibroblasts to migrate to the fracture site, induction of the proliferation of MSCs and fibroblasts, and induction of osteogenic differentiation of MSCs.

In conclusion, the present study revealed that CCNPs alone have limited efficacy in bone generation and the addition of A-PRF and/or AgNPs to CCNPs reduced its resorption rate compared to CCNPs alone as well as improved its osteogenic and osteoinductive properties by husting of new bone formation.

## Data Availability

Datasets supporting the conclusions of this article are included within the article.

## References

[1] Rodríguez-Merchán EC (2022). Bone healing materials in the treatment of recalcitrant nonunions and bone defects. Int. J. Mol. Sci..

[2] Ni N, Ge M, Huang R (2023). Thermodynamic 2D silicene for sequential and multistage bone regeneration. Adv. Healthc. Mater..

[3] Misk TN, Abd El-kawi M, Gadallah S, Elgohary E, Sharshar A (2020). Studies on A-PRF and A-PRF/coral powder for reconstruction of induced bone defects in dogs. J. Curr. Vet. Res..

[4] Fernandez de Grado G, Keller L, Idoux-Gillet Y (2018). Bone substitutes: A review of their characteristics, clinical use, and perspectives for large bone defects management. J. Tissue Eng..

[5] Wickramasinghe ML, Dias GJ, Premadasa KMGP (2022). A novel classification of bone graft materials. J. Biomed. Mater. Res. Part B Appl. Biomater..

[6] El-Bahrawy AA, El-Hamadan S, El-Ballal S, Sharshar A (2021). Comparative evaluation of bone regenerating capacity using nanocrystalline hydroxyapatite and coral composite: A canine model study. J. Curr. Vet. Res..

[7] Babuska V, Kasi PB, Chocholata P (2022). Nanomaterials in bone regeneration. Appl. Sci..

[8] Kumar VB, Khajuria DK, Karasik D, Gedanken A (2019). Silver and gold doped hydroxyapatite nanocomposites for enhanced bone regeneration. Biomed. Mater..

[9] Khajuria DK, Razdan R, Mahapatra DR (2015). Development, in vitro and in vivo characterization of zoledronic acid functionalized hydroxyapatite nanoparticle based formulation for treatment of osteoporosis in animal model. Eur. J. Pharm. Sci..

[10] Gadallah SM, Abd-Elkawi M, Misk TN, Sharshar AM (2022). The efficacy of nano-calcium carbonate derived from coral reefs and nano-silver to induce new bone formation in critical radial bone defect in rabbits: Radiological evaluation. J. Curr. Vet. Res..

[11] Khajuria DK, Kumar VB, Gigi D, Gedanken A, Karasik D (2018). Accelerated bone regeneration by nitrogen-doped carbon dots functionalized with hydroxyapatite nanoparticles. ACS Appl. Mater. Interfaces.

[12] Sun C-Y, Che Y-J, Shi-jin L (2015). Preparation and application of collagen scaffold-encapsulated silver nanoparticles and bone morphogenetic protein 2 for enhancing the repair of infected bone. Biotechnol. Lett..

[13] Zhao Y, Liu J, Zhang M (2020). Use of silver nanoparticle–gelatin/alginate scaffold to repair skull defects. Coatings.

[14] Chen F, Han J, Guo Z (2023). Antibacterial 3D-printed silver nanoparticle/poly lactic-co-glycolic acid (PLGA) scaffolds for bone tissue engineering. Materials (Basel).

[15] Qing T, Mahmood M, Zheng Y, Biris AS, Shi L, Casciano DA (2018). A genomic characterization of the influence of silver nanoparticles on bone differentiation in MC3T3-E1 cells. J. Appl. Toxicol..

[16] Hermida-Nogueira L, Blanco J, García Á, Greening DW, Simpson RJ (2023). Secretome profile of leukocyte-platelet-rich fibrin (L-PRF) membranes. Serum/plasma proteomics: Methods and protocols.

[17] Kargarpour Z, Panahipour L, Mildner M, Miron RJ, Gruber R (2023). Lipids of platelet-rich fibrin reduce the inflammatory response in mesenchymal cells and macrophages. Cells.

[18] Ghanaati S, Booms P, Orlowska A (2014). Advanced platelet-rich fibrin: a new concept for cell-based tissue engineering by means of inflammatory cells. J. Oral Implantol..

[19] Ravi S, Santhanakrishnan M (2020). Mechanical, chemical, structural analysis and comparative release of PDGF-AA from L-PRF, A-PRF and T-PRF-an in vitro study. Biomater. Res..

[20] Dare S, Bajaj P (2023). Evaluation of effectiveness of advanced platelet rich fibrin (A-PRF) with demineralized freeze-dried bone allograft (DFDBA) placed into fresh extraction sockets with immediate implant placement: A clinical and radiographic study. F1000Research.

[21] Hussein AI, Ab-Ghani Z, Che Mat AN, Ab Ghani NA, Husein A, Ab Rahman I (2020). Synthesis and characterization of spherical calcium carbonate nanoparticles derived from cockle shells. Appl. Sci..

[22] Amer SA, Nouh SR, Elkammar MH, Shalaby TI, Korittum AS (2018). Silver nanoparticles preparation and their effect on full-thickness skin wound healing in rabbit model. Alexandria J. Vet. Sci..

[23] Abd Elkawi M, Gadallah SM, Misk TN, Sharshar AM (2022). Potential effect of a novel composite (calcium carbonate nanoparticles/silver nanoparticles/advanced-platelet rich fibrin) on healing of critical bone defect in rabbits: radiographic evaluation. J. Curr. Vet. Res..

[24] Emery SE, Brazinski MS, Koka A, Bensusan JS, Stevenson S (1994). The biological and biomechanical effects of irradiation on anterior spinal bone grafts in a canine model. JBJS.

[25] Xuan Y, Li L, Zhang C, Zhang M, Cao J, Zhang Z (2023). The 3D-printed ordered bredigite scaffold promotes pro-healing of critical-sized bone defects by regulating macrophage polarization. Int. J. Nanomed..

[26] Xie C, Wang C, Wenwen Huang Y, Huang QL, Chengqiang Y, Yin D (2023). Recombinant human bone morphogenetic protein is a valid alternative to autologous bone graft for long bone non-unions: A systematic review and meta-analysis. Surgeon.

[27] Kungvarnchaikul I, Subbalekha K, Sindhavajiva PR, Suwanwela J (2023). Deproteinized bovine bone and freeze‐dried bone allograft in sinus floor augmentation: A randomized controlled trial. Clin. Implant Dent. Relat. Res..

[28] Moon YJ, Jeong S, Lee KB (2023). Bone morphogenetic protein 2 promotes bone formation in bone defects in which bone remodeling is suppressed by long-term and high-dose Zoledronic acid. Bioengineering.

[29] Soares LFF, Carrera TMI, de Oliveira Alves R, *et al*. Platelet-rich fibrin (PRF) membrane isolated and injectable-PRF-mixed particulate bovine bone graft in rattibiae non-critical defects healing (2023).

[30] Kargarpour Z, Panahipour L, Miron RJ, Gruber R (2022). Blood clots versus PRF: Activating TGF-β signaling and inhibiting inflammation in vitro. Int. J. Mol. Sci..

[31] Amiryaghoubi N, Fathi M, Barar J, Omidian H, Omidi Y (2023). Advanced nanoscale drug delivery systems for bone cancer therapy. Biochim. Biophys. Acta (BBA) – Mol. Basis Disease.

[32] Fadia P, Tyagi S, Bhagat S (2021). Calcium carbonate nano-and microparticles: synthesis methods and biological applications. 3 Biotech..

[33] Zalama E, Karrouf G, Rizk A, Salama B, Samy A (2022). Does zinc oxide nanoparticles potentiate the regenerative effect of platelet-rich fibrin in healing of critical bone defect in rabbits?. BMC Vet. Res..

[34] Luceri A, Francese R, Lembo D, Ferraris M, Balagna C (2023). Silver nanoparticles: review of antiviral properties, mechanism of action and applications. Microorganisms.

[35] Nie P, Zhao Y, Xu H (2023). Synthesis, applications, toxicity and toxicity mechanisms of silver nanoparticles: A review. Ecotoxicol. Environ. Saf..

[36] Ueda M, Yokota T, Honda M (2021). Regulating size of silver nanoparticles on calcium carbonate via ultrasonic spray for effective antibacterial efficacy and sustained release. Mater. Sci. Eng. C.

[37] Hill JM, Qi B, Bayaniahangar R (2019). Nanomaterials for bone tissue regeneration: updates and future perspectives. Nanomedicine.

[38] Shibahara K, Hayashi K, Nakashima Y, Ishikawa K (2022). Effects of channels and micropores in honeycomb scaffolds on the reconstruction of segmental bone defects. Front. Bioeng. Biotechnol..

[39] Zhang B, Su Y, Zhou J, Zheng Y, Zhu D (2021). Toward a better regeneration through implant-mediated immunomodulation: harnessing the immune responses. Adv. Sci..

[40] Isaksson H, Harjula T, Koistinen A (2010). Collagen and mineral deposition in rabbit cortical bone during maturation and growth: Effects on tissue properties. J. Orthop. Res..

[41] Mäkitaipale J, Sievänen H, Laitinen-Vapaavuori O (2018). Tibial bone density, cross-sectional geometry and strength in Finnish pet rabbits: a peripheral quantitative computed tomography study. Vet. Rec..

[42] Ou KL, Hou PJ, Huang BH (2020). Bone healing and regeneration potential in rabbit cortical defects using an innovative bioceramic bone graft substitute. Appl. Sci..

[43] Salih SI, Al-Falahi NH, Saliem AH, Abedsalih AN (2018). Effectiveness of platelet-rich fibrin matrix treated with silver nanoparticles in fracture healing in rabbit model. Vet. World.

[44] Hassibi H, Farsinejad A, Dabiri S (2020). Allogenic bone graft enriched by periosteal stem cell and growth factors for osteogenesis in critical size bone defect in rabbit model: histopathological and radiological evaluation. Iran J. Pathol..

[45] Chen X, Wang J, Yu L, Zhou J, Zheng D, Zhang B (2018). Effect of concentrated growth factor (CGF) on the promotion of osteogenesis in bone marrow stromal cells (BMSC) in vivo. Sci. Rep..

[46] Fang J, Shi R, Qi W, Zheng D, Zhu H (2023). Feasibility evaluation of the induced membrane technique with structural autologous strip bone graft management of phalangeal and metacarpal segmental defects using radiography. BMC Musculoskelet Disord..

[47] Zhang R, Lee P, Lui VCH (2015). Silver nanoparticles promote osteogenesis of mesenchymal stem cells and improve bone fracture healing in osteogenesis mechanism mouse model. Nanomed. Nanotechnol., Biol. Med..

